# Extracts from the Proceedings of the Medical Association of Georgia, for 1861

**Published:** 1866-06

**Authors:** 


*Extracts from the Proceedings of the Medical Association of Georgia, for* 1861.

The President having ordered a ballot for officers for the ensuing year, a ballot was taken, which resulted in the election of

Dr. J. T. Banks, of Griffin, President.
Dr. J. F. Alexander, of Atlanta, 1st Vice-President.
Dr. V. H. Taliaferro, of Columbus, 2d Vice-President.
Dr. A. G. Thomas, of Atlanta, Secretary.

AFTERNOON SESSION, 3 o'clock, P. M.

Minutes of forenoon read.

Reports from auxiliary societies being called for, Dr. B. B. Brown reported the formation of a society of physicians residing in the counties of Catoosa, Whitfield and Murray, called the State Line Medical Association, composed of fifteen members, holds monthly meetings, has adopted the Constitution and Code of Ethics of the Medical Association of Georgia, and desired to be admitted as an auxiliary to that body.

On motion of Dr. Coe, the Society was admitted as auxiliary.

Dr. J. G. Westmoreland, from committee appointed at last meeting to memorialize legislature, made a report, which, on motion of Dr. Coe, was received, and committee continued till next meeting.

Dr. Logan moved to defer action on the resolution with regard to permanent location of this Association till next meeting. After some discussion was carried.

On motion of Dr. Alexander, committee on revision of Constitution and By-Laws was continued, to report at next meeting.

On motion, committee on Medical Literature was discharged.

On motion of Dr. Simmons, committee on Finance was continued till next meeting.

Dr. O'Keefe moved that a committee of three be appointed to raise $100, to be distributed as prizes for the three best Essays presented to next meeting—$50 for the first, $30 for the second, and $20 for the third. Carried. Committee, Drs. O'Keefe, Taliaferro and Boyd.

On motion, the President appointed, as the committee to examine Prize Essays, Drs. H. Coe, A. Means, J. F. Alexander, H. W. Brown and T. C. H. Wilson.

On motion of Dr. Coe, a committee of three was appointed to take convenient time and procure the services of Orator and alternate for next meeting. Committee, Drs. Coe, Dannelly and Brown.

Dr. O'Keefe offered the following, which, on motion of Dr. Logan, was adopted:

*Resolved*, That the committee on Prize Essays be instructed, in awarding prizes, to give preference to subjects of practical interest to the profession, and to award the prizes publicly, during the session of the Association, in cash or its equivalent, at the discretion of the committee.

On motion, the President was required to appont at his convenience a committee of arrangements.

